# HPV DNA testing in population-based cervical screening (VUSA-Screen study): results and implications

**DOI:** 10.1038/bjc.2011.581

**Published:** 2012-01-17

**Authors:** D C Rijkaart, J Berkhof, F J van Kemenade, V M H Coupe, L Rozendaal, D A M Heideman, R H M Verheijen, S Bulk, W Verweij, P J F Snijders, C J L M Meijer

**Affiliations:** 1 Department of Pathology, VU University Medical Center, PO Box 7057, 1007 Amsterdam, The Netherlands; 2 Department of Epidemiology and Biostatistics, VU University Medical Center, Amsterdam, The Netherlands; 3 Division of Woman and Baby, Gynaecological Oncology, University Medical Center, Utrecht, The Netherlands; 4 Department of Medical Genetics, University Medical Center, Utrecht, The Netherlands; 5 SALTRO, Primary Health Care Laboratory, Utrecht, The Netherlands

**Keywords:** cytology, human papillomavirus, hybrid capture, cervical intraepithelial neoplasia, early detection of cancer

## Abstract

**Background:**

Human papillomavirus (HPV) testing is more sensitive than cytology for detecting high-grade cervical intraepithelial neoplasia (CIN). We evaluated the performance of high-risk HPV (hrHPV) testing in routine screening.

**Methods:**

In all, 25 871 women (29–61) enrolled in our population-based cohort study were offered both cytology and hrHPV testing. High-risk HPV-positive women with normal cytology and an age-matched subcohort of hrHPV-negative women with normal cytology were invited for repeat testing after 1 and/or 2 years and were referred for colposcopy if they presented with abnormal cytology and/or a positive hrHPV test. The hrHPV-positive women with borderline or mild dyskaryosis (BMD) and all women with moderate dyskaryosis or worse (>BMD) were directly referred for colposcopy. Women with BMD and an hrHPV-negative test were advised to repeat cytology at 6 and 18 months and were referred for colposcopy if the repeat cytology test was abnormal. The main outcome measure was CIN grade 3 or worse (CIN3+). Results were adjusted for non-attendance at repeat testing.

**Results:**

The hrHPV-positive women with abnormal cytology had a CIN3+ risk of 42.2% (95% confidence interval (CI): 36.4–48.2), whereas the hrHPV-positive women with normal cytology had a much lower risk of 5.22% (95% CI: 3.72–7.91). In hrHPV-positive women with normal cytology, an additional cytology step after 1 year reduced the CIN3+ risk to only 1.6% (95% CI: 0.6–4.9) if the repeat test was normal. The CIN3+ risk in women with hrHPV-positive normal cytology was higher among women invited for the first time (29–33 years of age) (9.1% 95% CI: 5.6–14.3) than among older women (3.0% 95% CI: 1.5–5.5).

**Conclusion:**

Primary hrHPV screening with cytology triage in women aged ⩾30 years is an effective way to stratify women on CIN3+ risk and seems a feasible alternative to cytological screening. Repeat cytology after 1 year for hrHPV-positive women with normal cytology is however necessary before returning women to routine screening.

Cytological screening has reduced the incidence and mortality of cervical cancer in countries with organised screening programmes (Arbyn *et al*, 2009). However, cytological screening offers a suboptimal prevention against cervical cancer as cytology has a limited sensitivity for high-grade cervical intraepithelial neoplasia (CIN) (van den Akker-van Marle *et al*, 2002; Cuzick *et al*, 2006). Many studies conducted by combined high-risk human papillomavirus (hrHPV) and cytology testing have revealed that testing for hrHPV results in a much higher sensitivity for high-grade CIN and cervical cancer (CIN2+) than cytology (Cuzick *et al*, 2006; Bulk *et al*, 2007; Bulkmans *et al*, 2007; Mayrand *et al*, 2007; Naucler *et al*, 2007; Ronco *et al*, 2008, 2010).

Although the data collected so far are in favour of implementing hrHPV testing in primary screening there is still debate about the management of hrHPV-positive women and about the screening ages at which hrHPV testing would be most beneficial. In young women, the prevalence of hrHPV is high and as a consequence the management of hrHPV-positive women may be complicated.

The higher number of positive primary screening tests in this age group may lead to adverse effects of screening if more unnecessary follow-up tests and colposcopy referrals are made. This is of particular importance for these women of reproductive age, because it has been shown that the rate of serious obstetrical complications, such as preterm deliveries, low birth weight and premature rupture of the membranes, is increased after excisional treatments for precancerous lesions (Kyrgiou *et al*, 2006).

Furthermore, young women may have a disproportional high number of regressive CIN2 lesions. Ronco *et al* (2010) have shown that primary hrHPV screening is particularly effective for women 35 years or older, whereas in younger women hrHPV screening would lead to overdiagnosis of regressive CIN2. On the other hand, Bulkmans *et al* (2007) demonstrated that in women between 30 and 60 years the total number of CIN2+ lesions over two screening rounds was equal in both the hrHPV plus cytology arm and the cytology only arm, indicating that there is no CIN2 overdiagnosis in the hrHPV plus cytology arm. Instead, more high-grade lesions were detected earlier in the hrHPV plus cytology arm than in the control arm. This indicates that in this age category hrHPV testing detects non-regressing, clinically relevant CIN2+ lesions earlier than cytology and suggests that primary hrHPV screening in women of ⩾30 years is feasible.

To evaluate for the Dutch cervical screening programme the effectiveness of implementing hrHPV testing and to assess future implementation issues, we set up the VUSA-Screen study (Vrije Universiteit Medical Centre-*Sa*ltro laboratory population-based cervical screening). The study was carried out within the setting of a routine cervical screening programme. We present the main results of this cohort study in which 3-year follow-up results were related to baseline hrHPV testing and cytology testing to find an optimal primary screening method. Special attention was given to the question whether hrHPV testing should be offered in combination with cytology or as a sole primary screening instrument. In addition, we study how hrHPV-positive women should be managed. Finally, we examine at what age (at 30 years or at older age) it would be most beneficial to start hrHPV testing.

## Patients and methods

### Patients and procedures

The VUSA-Screen study is a cohort study within the setting of the Dutch population-based cervical screening programme designed to evaluate the effectiveness of combined cervical cytology screening with hrHPV testing by the HC2 hybridisation assay (Qiagen, Gaithersburg, MD, USA). In the Netherlands, women are invited for cervical cancer screening at 5-year intervals starting in the year in which they reach the age of 30 and with the last invitation in the year in which they turn 60 (age range, 29–61 years). The study was carried out in the province of Utrecht in the Netherlands among women who were invited for the regular cervical screening programme between October 2003 and August 2005. The design of the study, including exclusion criteria, has been described previously (Rijkaart *et al*, 2010). All participants gave written informed consent. The VUSA-Screen study was approved by the Ministry of Public Health (2002/02-WBO; ISBN-10: 90-5549-452-6) and registered in the trial register (NTR215, ISRCTN64621295).

From all participants, a conventional cytological smear was taken with a cytobrush (Rovers, Oss, The Netherlands). After preparation of the smear on a glass slide, the brush was placed in a vial containing 1 ml UCM (Universal Collection Medium; Qiagen) for hrHPV testing. Cervical cytology results were reported, blinded to the hrHPV testing results, according to the CISOE-A classification, which is routinely used in the Netherlands and can be easily converted into the 2001 Bethesda system (Bulk *et al*, 2004). Cytological results were grouped as normal, borderline or mild dyskaryosis (BMD), and moderate dyskaryosis or worse (>BMD). In the 2001 Bethesda system, BMD corresponds to atypical squamous cells of undetermined significance, atypical squamous cells, cannot exclude high-grade squamous intraepithelial lesions, or low-grade squamous intraepithelial lesions. Moderate dyskaryosis or worse corresponds to high-grade squamous or glandular intraepithelial lesions.

High-risk HPV testing was performed by HC2 high-risk HPV DNA test in an automated format on a rapid capture system according to manufacturer's instructions (Qiagen). This test uses a cocktail probe to detect 13 high-risk HPV types: 16, 18, 31, 33, 35, 39, 45, 51, 52, 56, 58, 59 and 68. Samples with HC2 outcome of ⩾1 RLU/CO were considered as hrHPV positive. HC2-positive samples were tested with GP5+/6+-PCR-EIA (Jacobs *et al*, 1997), and all specimens tested positive by GP5+/6+-PCR-EIA were typed by reverse line blotting (van den Brule *et al*, 2002).

**Figure 1:**
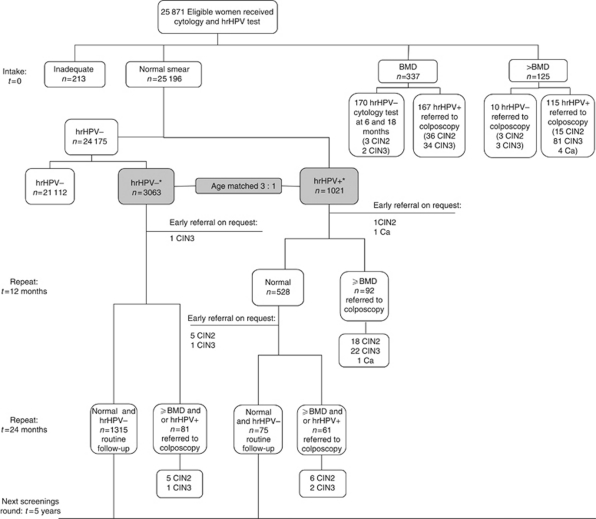
Flowchart of the screening profiles of women in the VUSA-Screen study. hrHPV=high-risk human papillomavirus; hrHPV+=positive hrHPV test; hrHPV−=negative hrHPV test; BMD=borderline or mild dyskaryosis; >BMD=moderate dyskaryosis or worse; CIN=cervical intraepithelial neoplasia (grade 2 or 3); Ca=cervical carcinoma. ^*^The baseline hrHPV test results of these matched women were blinded.

Women with BMD or worse were informed about the hrHPV test result. The HrHPV-positive women with BMD and all women with >BMD were directly referred for colposcopy (Figure 1). Women with BMD and a negative hrHPV test were offered cytology at 6 and 18 months and referred if cytology was abnormal (threshold BMD) at one of these occasions.

In the women with normal cytology at baseline, a subcohort was selected. In this subcohort, all (*n*=1021) hrHPV-positive women as well as a subset of hrHPV-negative, cytologically normal women (*n*=3063) were included. To select the hrHPV-negative women, each hrHPV-positive woman was matched to three randomly chosen hrHPV-negative women of the same age. Women were not informed about the hrHPV test result. Women with normal cytology and hrHPV-positive test were offered cytology and a blinded hrHPV test at 12 months and combined hrHPV and cytology testing at 24 months. Women were referred at 12 months if cytology was abnormal and at 24 months if cytology was abnormal and/or the hrHPV test was positive.

Women in the subcohort with normal cytology and hrHPV-negative test were invited for repeat testing with both tests at 24 months. These women were referred at 24 months if cytology was abnormal and/or the hrHPV test was positive. Women with normal cytology who were not included in the subcohort were recalled at the next screening round after 5 years as part of the routine screening programme.

Of the women who were referred to a gynaecologist for colposcopy, colposcopy-directed biopsies were taken from suspicious areas of the cervix, according to standard procedures in the Netherlands (Hopman *et al*, 2000). Biopsy results were reported as normal, CIN1, 2, or 3, or as invasive cancer, according to the international criteria (Anderson, 1995). Cytology and histology results were retrieved from the nationwide network and registry of histopathology and cytopathology (PALGA, Bunnik, The Netherlands).

### Statistical analysis

The primary outcome measure of the study was histologically confirmed CIN3+, detected cumulatively within 3 years after baseline. A secondary outcome was cumulatively detected CIN2+. In the calculations of the number of CIN3+ and CIN2+ lesions, also cases of cervical adenocarcinoma and cervical adenocarcinoma *in situ* were included.

Separate CIN3+ and CIN2+ risks were calculated for hrHPV, cytology and age-specific strata. The risks were adjusted for non-attendance at repeat testing. Non-attendance rates at 12 and 24 months may depend on previous screening test results and were read from flow charts (Figure 1).

The sensitivity and specificity of the hrHPV test and cytology were adjusted for non-attendance at repeat testing by writing them as functions of stratum-specific CIN3+ or CIN2+ risks (Kulasingam *et al*, 2002). Ninety-five percent confidence intervals (CIs) for the CIN3+ and CIN2+ risks and for the sensitivities and specificities were obtained from Bayesian posterior distributions. To compute posteriors, Beta (0.5,0.5) priors were imposed on the probabilities of moving from one box to another box in flowchart (Figure 1). The posterior intervals were computed via simulation. The posterior intervals may become narrow when one or more of the point estimates of the probabilities equal 0 (or 1). We accounted for this by imposing a point prior at 0 (or 1) and recomputed the Bayesian posterior interval. The reported CIs are unions of the original and recomputed posterior intervals. This approach has reasonable frequentist properties when estimating a proportion (Cai, 2005).

Analyses were done with SPSS version 15.0 (LEAD Technologies Inc., Haddonfield, NJ, USA), Excel (Microsoft Corporation, Redmond, WA, USA), and Matlab version 7.9 (The Mathworks Inc., Natick, MA, USA).

## Results

Of the 25 871 women recruited for the VUSA-Screen study, 25 658 (99. 2%) had adequate baseline cytology and hrHPV HC2 test. The median age of participating women was 44.0 years (range 29–61 years). Among the women with adequate cytology, 97.4% had a normal result, 1.3% had BMD and 0.5% had >BMD. The proportion of women with hrHPV infection(s) was 4.1% in women with normal cytology, 49.6% in women with BMD and 92.0% in women with >BMD. Overall, 5.1% (1303 out of 25 658) of the women tested hrHPV positive by HC2. In women with BMD and negative hrHPV result, the overall compliance to repeat testing was 86.5%. In the subcohort of women with normal cytology, compliance to repeat testing was similar in the blinded hrHPV-positive and hrHPV-negative group (61.8% and 59.7%, respectively, *P*=0.237). For women with normal cytology at baseline with follow-up, the histology follow-up at 24 months showed a higher referral rates after abnormal cytology than after an hrHPV-positive, cytologically normal test result (57.0% *vs* 21.1%, respectively). Among women who attended at repeat testing, the average time to the first follow-up test was 15.0 months with a standard deviation of 4.7 months. The follow-up time ranged from 1.3 to 28.6 months.

**Figure 2:**
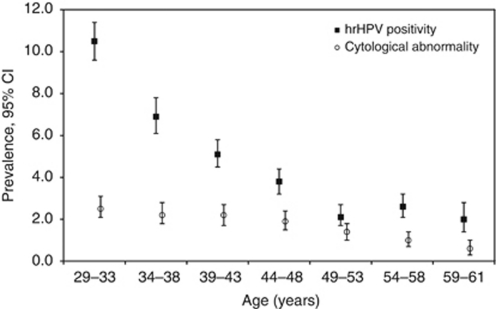
Age-specific prevalence of hrHPV positivity and cytological abnormalities in women of the VUSA-Screen study. hrHPV=high-risk human papillomavirus; cytological abnormalities=borderline or mild dyskaryosis or worse (⩾BMD).

We evaluated hrHPV prevalence in seven age groups corresponding to the screening rounds. We found the highest hrHPV prevalence among women between 29 and 33 years of age who were invited for the first time (10.5% 95% CI: 9.6–11.4%). As the age increased, hrHPV prevalence decreased until age 49 years. The hrHPV prevalence in women aged 59–61 years was 2.0% (95% CI: 1.5–2.8% Figure 2). Women aged 29–33 years showed a significantly higher hrHPV prevalence (10.5% 95% CI: 9.6–11.4%) than women aged 34–61 (4.0% 95% CI: 3.7–4.3%) (*P*<0.001). Among women with adequate cytology, 1.8% (95% CI: 1.6–2.0%) had an abnormal result (⩾BMD). The highest proportion of abnormal cytology was found in women aged 29–33 years (i.e., 2.5% 95% CI: 2.1–3.1%) and the lowest proportion was found in women aged 59–61 years (0.6% 95% CI: 0.3–1.0%).

The histological follow-up results in relation to baseline cytology and hrHPV test results, stratified by two age groups, are presented in Table 1. Among women with normal cytological results, the proportion of CIN3+ cases was 2.6% (27 out of 1021) if the hrHPV test was positive and 0.07% (2 out of 3063) if the hrHPV test was negative. For women with abnormal cytology, the proportion of CIN3+ cases was 42.2% (119 out of 282) if the hrHPV test was positive and 2.8% (5 out of 180) if the hrHPV test was negative.

Of 1021 women with normal cytology and a positive hrHPV test at baseline, 92 women had abnormal cytology at 12 months follow-up of whom 76 (82.6%) tested hrHPV positive, 6 (6.5%) tested hrHPV negative and 10 (10.9%) women had an unknown hrHPV status. Of the women with normal cytology and a positive hrHPV test at baseline, 528 had normal cytology at 12 months follow-up of whom 224 (42.4%) tested hrHPV positive, 219 (41.5%) tested hrHPV negative and 84 (15.9%) had an unknown hrHPV status. The attendance at 24 months was comparable for women with normal cytology and hrHPV-positive test at 12 months (14 women with normal cytology and hrHPV-negative test and 52 women with abnormal cytology and/or hrHPV-positive test) and women with normal cytology and hrHPV-negative test (52 women with normal cytology and hrHPV-negative test and 6 women with abnormal cytology and/or hrHPV-positive test).

The absolute and relative sensitivity and specificity of cytology and hrHPV testing for detection of CIN3+ and CIN2+ are presented in Table 2. The sensitivity of hrHPV testing for CIN3+, adjusted for non-attendance at repeat testing, was 1.42-fold higher than the sensitivity of cytology (91.9% *vs* 64.6%) at the cost of a lower specificity (95.6% *vs* 98.7%). The sensitivity of hrHPV testing for CIN2+ was 1.63-fold higher than cytology (82.0% *vs* 50.5%); however, the specificity was 0.97 fold lower (96.0% *vs* 98.9%).

**Figure 3:**
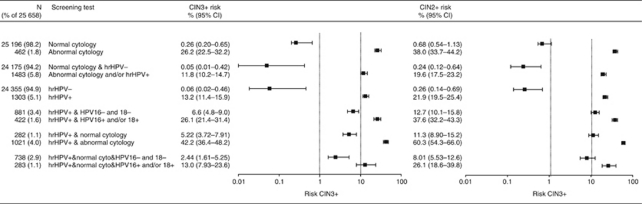
Cumulative 3-year risk of CIN3+ and CIN2+ stratified by cytology, hrHPV status and HPV16/18 genotype test results adjusted for non-attendance at repeat testing. Abbreviations: hrHPV=high-risk human papillomavirus; hrHPV+=positive hrHPV test; hrHPV−=negative hrHPV test; abnormal cytology, borderline or mild dyskaryosis or worse (⩾BMD); CI=confidence interval; CIN=cervical intraepithelial neoplasia (grade 2 or 3 or higher).

The cumulative 3-year CIN3+ and CIN2+ risks, adjusted for non-attendance at repeat testing, are presented in Figure 3. The CIN3+ risk was markedly lower in women negative for hrHPV (0.06% 95% CI: 0.02–0.46%) than in women with negative cytology (0.26%, 95% CI: 0.20–0.65%). There was only a small, non-significant difference in CIN3+ risks between women with negative results on both tests (0.05% 95% CI: 0.01–0.42) and women negative for hrHPV only (0.06% 95% CI: 0.02–0.46). Women with abnormal cytology (⩾BMD) result had a CIN3+ risk of 26.2% (95% CI: 22.5–32.2) and those with an hrHPV-positive test had a risk of 13.2% (95% CI: 11.4–15.9). The highest CIN3+ risk (i.e., 42.2% 95% CI: 36.4–48.2) was found in hrHPV-positive women who had abnormal cytology. The HPV16/18 genotyping at baseline showed that hrHPV-positive women for other types than HPV16/18 still had a CIN3+ risk of 6.6% (95% CI: 4.8–9.0). The HPV16+ and/or 18+ women had a CIN3+ risk of 26.1% (95% CI: 21.4–31.4) (Figure 3).

The majority of the hrHPV-positive women had normal cytology and those women still had a CIN3+ risk of 5.22% (95% CI: 3.72–7.91). The HPV16/18 genotyping of hrHPV-positive women at baseline did not result in sufficient low risks for a screenings programme with 5 years interval. Women with hrHPV-positive normal cytology and HPV16 and/or HPV18-positive test had a CIN3+ risk of 13.0% (7.93–23.6), whereas women who tested hrHPV positive for other high-risk types had a much lower risk of 2.44% (95% CI: 1.61–5.25). We also evaluated a baseline triage and follow-up strategy for hrHPV-positive women. Baseline cytology triage followed by repeat cytology testing at 1 year showed that the CIN3+ risk reduced to only 1.6% (95% CI: 0.6–4.9) in women with normal cytology at the repeat test. In women with abnormal cytology at the repeat test, the CIN3+ risk was 25.0% (95% CI: 16.6–35.1). This CIN3+ risk is comparable to the CIN3+ risk of hrHPV-positive women with abnormal cytology at baseline.

Analysis using CIN2+ as outcome measure found comparable results, albeit with higher absolute risks. The cumulative 3-year CIN2+ risk was 0.26% (95% CI: 0.14–0.69%) among hrHPV-negative women and 0.68% (95% CI: 0.54–1.13%) among women with negative cytology. The CIN2+ risk was similar for women with negative results on both tests (0.24% 95% CI: 0.12–0.64%) and women negative for hrHPV only (0.26% 95% CI: 0.14–0.69%). The hrHPV-positive women with normal cytology had a CIN2+ risk of 11.3% (95% CI: 8.90–15.2) and hrHPV-positive women with normal cytology and negative for HPV16/18 genotyping had a CIN2+ risk of 8.01% (95% CI: 5.53–12.6).

When stratifying hrHPV-positive women into two age groups, there was a borderline non-significant difference in CIN3+ risk (adjusted for non-attendance at repeat testing) between women ⩾34 years of age and women invited for the first time (aged 29–33 years) (relative risk (RR) 0.78 95% CI: 0.52–1.15). The RR for CIN2+ was 0.87 (95% CI: 0.64–1.18). In hrHPV-positive women with normal cytology, the CIN3+ risk was significantly lower in women aged ⩾34 years (4.0; 95% CI: 2.3–6.6) than in women aged 29–33 years (10.9% 95% CI: 5.9–19.2). The corresponding RR was 3.02 (95% CI: 1.39–7.07). The CIN2+ risk was 10.7 (95% CI: 7.3–15.3) in women with normal cytology and age ⩾34 years, 16.6% (95% CI: 10.7–25.3) in women with normal cytology and age 29–33 years, and the corresponding RR was 0.65 (95% CI: 0.37–1.14). When stratifying hrHPV-positive women with abnormal cytology into two age groups, no risk difference between the older and younger age group was observed for CIN3+ and CIN2+ (RR CIN3+ 1.03; 95% CI: 0.78–1.42; RR CIN2+ 0.95; 95% CI: 0.79–1.18).

## Discussion

Implementation of hrHPV testing as a primary screening instrument in cervical screening is still under debate. The presented study enables us to examine three implementation issues in more detail. First, whether hrHPV testing should be offered in combination with cytology or as a sole primary screening instrument. Second, how hrHPV-positive women should be managed. Third, the screening ages at which hrHPV testing would be most beneficial.

In our study, hrHPV testing has a 27.3% higher sensitivity but a 3.1% lower specificity than cytology for detection of CIN3+. For CIN2+, these figures were 31.5% and 2.9%, respectively. These results are in line with other screening studies (Kotaniemi-Talonen *et al*, 2005; Arbyn *et al*, 2006; Ronco *et al*, 2006a, 2006b; Mayrand *et al*, 2007; Naucler *et al*, 2007; Leinonen *et al*, 2009; Giorgi-Rossi *et al*, 2011) which have demonstrated that hrHPV testing is superior to cytology in terms of sensitivity but not in terms of specificity. Women with a negative hrHPV test were found to have a very low risk of an underlying or incipient high-grade CIN lesion and their CIN3+ risk is not markedly lower after ascertainment that cytology is normal. Therefore, from a health-economic perspective, cervical screening with a primary, stand-alone hrHPV test seems preferable. Similar recommendations have been made based on the recent cost-effectiveness studies (Goldhaber-Fiebert *et al*, 2008).

The hrHPV testing in primary screening creates a clinical dilemma for the management of hrHPV-positive women. These women are at mildly but significant risk of CIN3+ (13.2%). However, referring all hrHPV-positive women to colposcopy may result in overdiagnosis and overtreatment (Ronco *et al*, 2008). In current cytological screening practice, women with a BMD smear, having a CIN3+ risk of 6.4% (Rijkaart *et al*, 2010), are also not immediately referred for colposcopy but are advised to repeat cytology testing after 6 and 18 months (Hanselaar, 2002).

In the present study, we have used cytology as a triage tool to identify women at high risk for CIN3+ among the hrHPV-positive women. Women with an hrHPV-positive test and abnormal cytology had a CIN3+ risk of 42.2% and obviously need immediate colposcopy. On the other hand, hrHPV-positive women with normal cytology have a low, but still non-negligible CIN3+ risk of 5.2%. This risk is too high to delay follow-up to the next screening round (in the Netherlands 5 years) but too low to refer them for immediate colposcopy. Therefore, hrHPV-positive women with normal cytology at baseline require further triage testing and/or follow-up. In our study, women were retested after 1 year by means of cytology and after 2 years by both cytology and hrHPV. An analysis of the repeat testing results showed that the decision either referral to colposcopy or return to routine screening can be made after 1 year on the basis of one repeat cytological test. The CIN3+ risk after 1-year normal cytology was only 1.6%. This risk is similar to the CIN3+ risk of women with BMD at baseline and normal cytology at 6 and 18 months follow-up (1.2%), which is presently accepted in the Netherlands. Furthermore, the CIN3+ risk of 1.6% is also below the CIN3+ risk threshold proposed by Castle *et al* (2007) (2%) to justify no further follow-up. The CIN3+ risk after 1-year abnormal cytology was 25% and high enough to warrant referral for colposcopy.

Based on the present data, one may ask what the results are of other triage algorithms for HPV-positive women. This *post hoc* analysis on data of the present study is beyond the scope of this paper but has been presented in a separate paper (Rijkaart *et al*, 2011b).

We observed in our cohort study that the CIN3+ and CIN2+ detection rate in hrHPV-positive women was similar for women invited for cervical screening for the fist time (age 29–33 years) and for older women (⩾34 years). The same accounts for hrHPV-positive women with abnormal cytology. However, the CIN3+ detection rate in hrHPV-positive women with normal cytology was significantly higher among younger women (29–33) than among older women (⩾34). Ronco *et al* (2010) found in the hrHPV arm of women between 25 and 34 years a substantially higher proportion of CIN2 lesions than in women ⩾35 years. This coincided with an increase in detection of CIN2+ over two screening rounds compared with women ⩾35 years. It was argued that under the CIN2+ lesions detected in women younger than 35 years in the first round, a disproportionate number of regressive CIN2 lesions were present. We argue that such a potential age-related overdiagnosis does not occur in women ⩾30 years of age.

First, in the POBASCAM study the CIN2+/CIN3+ baseline detection in women over 30 years of age was higher in the hrHPV testing arm than in the cytology arm but over two screening rounds (interval 5 years), the CIN2+/CIN3+ detection rates were similar in both arms (Bulkmans *et al*, 2007). These data indicate that the increased detection of CIN2+/3+ lesions in the hrHPV arm at baseline in women over 30 years of age does not lead to overdiagnosis of regressive CIN2+ lesions but that the lesions are merely detected earlier and non-regressive, clinically relevant (Bulkmans *et al*, 2007).

In addition, in present study the CIN3+ and CIN2+ risk was similar in women invited for the first time (29–33 years) and in women ⩾34 years. Moreover, in hrHPV-positive women with normal cytology, the CIN3+ risk was higher in women invited for the first time than in older women. A possible explanation is that hrHPV infections detected in women invited for the first time may have persisted for many years before being identified by screening and therefore more likely to have developed into high-grade lesions. These results are in line with published data from the Guanacaste cohort (Rodriguez *et al*, 2010). In addition, we recently showed that the detection rate of CIN3+ and CIN2+ did not differ between women aged 29–33 years and women ⩾34 years (Rijkaart *et al*, 2011a). Moreover, this study indicates that hrHPV testing in women aged 29–33 years does not result in excessive diagnosis of regressive lesions.

### Limitations and strengths of the study

A limitation of our study is that women with normal cytology were not informed about the hrHPV status at baseline. This concealment was necessary to maximise attendance at repeat testing among hrHPV-negative women with normal cytology. The repeat testing attendance rate in women with normal cytology was 61.8% in hrHPV-negative and 59.7% in hrHPV-positive women. The attendance at repeat testing has been shown to be particularly poor after a cytologically normal test (Kitchener *et al*, 2009). The attendance rate of hrHPV-positive women in the present study might have been higher if women had been informed about their hrHPV test result.

We observed a higher percentage of histology reports after referral on the basis of abnormal cytology than after an hrHPV-positive, cytologically normal test result. This difference may be related to anticipated association between biopsy rate and colposcopic image of the cervix. If adjusted for, the effect of hrHPV testing on CIN3+ will be somewhat higher than the effect reported in this study. In this regard, several studies have indicated that the effect of hrHPV testing will be higher when a blind biopsy is carried out in women with a normal colposcopic impression (Pretorius *et al*, 2004).

Another limitation of our study may be the use of a subjective test such as cytology as a triage test for hrHPV-positive women. However, Leinonen *et al* (2009) reported that the influence of knowing the hrHPV results in reading cytology was small. In this context, it is expected that in the near future molecular biomarkers can be used as objective triage tests of hrHPV-positive women. Suitable candidate novel biomarkers such as HPV mRNA (Molden *et al*, 2005), methylation markers (Overmeer *et al*, 2008, 2009) or genotyping (Cuschieri *et al*, 2004) might further enhance the efficacy of screening with hrHPV DNA.

A strong point of our study is that this study is population based and is integrated in the regular screening programme of eligible women aged 29–61 years. The differences in sensitivity between hrHPV screening and cytological screening are slightly overestimated because in the practice of screening some women will not attend repeat testing after an hrHPV-positive test. Nevertheless, a higher attendance at repeat testing is to be expected once the implication of a positive hrHPV test is well communicated to the women and hrHPV screening becomes routine (Franco, 2009).

## Conclusions

Although cytology adds little to the reassurance from a negative hrHPV test against high-grade lesions, it is a very useful risk stratifier in hrHPV-positive women and results in a feasible screening algorithm. We showed that repeat cytology testing after 1 year for hrHPV-positive women with normal cytology at baseline is critical for maximising the benefits of primary hrHPV testing in routine cervical cancer screening.

## Figures and Tables

**Table 1:** Three-year cumulative histology outcome by age, baseline cytology and hrHPV results

**Age**	**Cytology**	**HrHPV**	**Total**	**CIN0/1**	**CIN2**	**CIN3**	**AdCa**	**SCC**	**CIN3+**	**CIN2+**
Invited for the first time (29–33 years)	Normal in subcohort	HrHPV+	367	15	11	12	1	0	13	24
		HrHPV−	1099	7	4	1	0	0	1	5
	Abnormal	HrHPV+	85	19	18	34	0	1	35	53
		HrHPV−	25	0	3	1	0	0	1	4
Others (34–61 years)	Normal in subcohort	HrHPV+	654	47	19	13	0	1	14	33
		HrHPV−	1964	24	1	1	0	0	1	2
	Abnormal	HrHPV+	197	56	33	81	1	2	84	117
		HrHPV−	155	18	3	4	0	0	4	7

Abbreviations: AdCa=adenocarcinoma; CIN=cervical intraepithelial neoplasia; hrHPV=high-risk human papillomavirus; hrHPV+=positive hrHPV test; hrHPV−=negative hrHPV test; abnormal cytology=borderline or mild dyskaryosis or worse (⩾BMD); normal in subcohort=a cohort of women with normal cytology and hrHPV− was age matched to hrHPV-positive women with normal cytology; SCC=squamous cell carcinoma.

Women in the subcohort were invited for combined testing at 24 months and referred if cytology was abnormal and/or the hrHPV test was positive.

**Table 2:** Absolute and relative sensitivity and specificity of hrHPV testing *vs* cytology, adjusted for non-attendance at repeat testing

**Screening test** a	**End point CIN3+**	**End point CIN2+**
	**(95% CI)**	**(95% CI)**
*Sensitivity*		
hrHPV	91.9% (61.0–96.7)	82.0% (62.9–89.6)
Cytology	64.6% (43.3–73.1)	50.5% (38.4–58.0)
		
*Specificity*		
hrHPV	95.6% (95.3–95.8)	96.0% (95.7–96.3)
Cytology	98.7% (98.5–98.8)	98.9% (98.7–99.0)
		
*Relative sensitivity*		
hrHPV *vs* cytology	1.42 (1.19–1.67)	1.63 (1.40–1.89)
		
*Relative specificity*		
hrHPV *vs* cytology	0.969 (0.966–0.971)	0.971 (0.968–0.974)

Abbreviations: hrHPV=high-risk human papillomavirus; CI=confidence interval; CIN=cervical intraepithelial neoplasia (grade 2 or 3 or higher).

a Cytology positivity was defined as a result of borderline or mild dyskaryosis or worse (⩾BMD).
